# Extending research impact by sharing maker information

**DOI:** 10.1038/s41467-023-41886-3

**Published:** 2023-10-04

**Authors:** Larry L. Howell, Terri Bateman

**Affiliations:** Brigham Young University, Provo, UT USA

**Keywords:** Science in culture, Mechanical engineering, Research management, Education

## Abstract

The availability of maker resources such as 3D printers, makerspaces, and public repositories enable researchers to share information with research peers, educators, industry, and the general public. This broadens the impact of research and inspires its extension and application.

While peer-reviewed publications are the gold standard for sharing scientific research results, many researchers, and their sponsors, also have a desire for their work to have an influence beyond their peers. Dissemination of new research usually involves translating technical results into forms more accessible to broader society. Valuable forms of translation include working with the popular media^1^, commercializing research results, creating exhibitions, posting on social media or lab websites, and sharing videos. The advance of fabrication resources capable of creating sophisticated geometries (particularly, but not exclusively, 3D printing) is making it viable to engage broader communities in ways not previously possible. Sharing maker information, such as 3D printer files and fabrication instructions, can be a powerful and efficient way to increase the impact of research among peers, application in industry, learning in education, and influence in society.

The making culture, where individuals and communities collaborate to create, use, and reuse information to make hardware, has been fueled by increased availability of manufacturing tools, the advent of makerspaces, the growth of an open-source culture, and the ease of sharing information, among other factors^2,3^. Emerging technologies and practices^4^ empower people to collaborate, create, and turn creative and futuristic ideas into reality^5^. The COVID-19 global pandemic exemplifies the benefits of engaging the maker community by using open-source design-sharing platforms to enhance collaboration, continuous development, and design dissemination of critical personal protective equipment (PPE)^6,7^. Just as the open-source software movement can result in software code competitive with that done professionally^8^, the open-source hardware movement is breaking down barriers as maker technology becomes more accessible.

Sharing maker information can help advance science in ways that individual labs cannot do alone. Interestingly, it is also part of a “virtuous cycle”: sharing resources increases visibility and builds credibility by demonstrating impact, which in turn helps relationships with peers and organizations that support the work, which in turn facilitates future work.

## Levels of engagement

We classify three levels of engagement ranging from more focused to more general audiences: (a) Research: sharing with research peers; (b) Applied: sharing with those who can apply the work, such as industry and educators; and (c) Society: sharing with the general public. This structure is based on our experiences working in compliant mechanisms and is one way to describe the various audiences, but there are alternative routes that can be taken between levels depending on the project intent and complexity of the information shared.

**Fig. 1:**
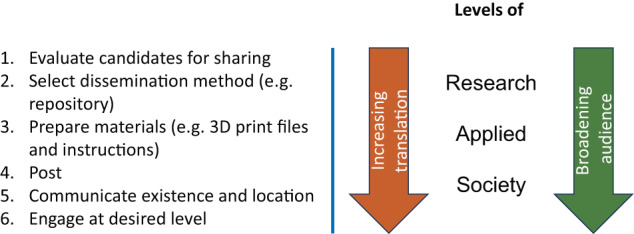
Steps for sharing and levels of engagement. Sharing maker files follows straightforward steps. Progressing through levels of engagement from research peers to those who apply the work (e.g., educators and industry), to general society often results in increasing translation of technical jargon but broadens the audience of the work.

**Fig. 2:**
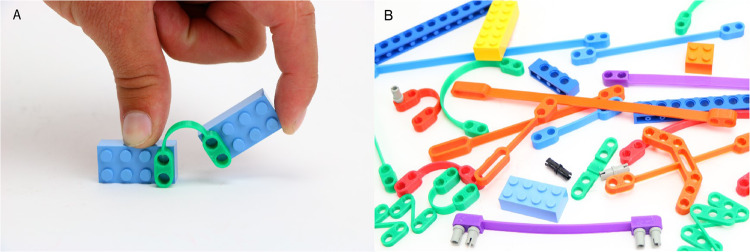
A simple illustrative example. **A** A “FlexLinks” component compatible with commercial building blocks and used to prototype compliant mechanisms, and **B** an illustration of a larger set of FlexLinks.

Here, we take an early effort from our lab as an illustrative example to discuss the progress from sharing files with research peers, to making them available to educators, industry, and broader society (see Fig. 1). Over ten years ago, before the start of a wider maker culture in academia and before an international movement on open science hardware, we created sets of “FlexLinks” (see Fig. 2) for quick turn-around proof-of-concept prototyping of compliant mechanisms^9^ (https://compliantmechanisms.byu.edu/). FlexLinks can be combined with commercially available building block components (e.g., LEGO®) to quickly build a wide range of simple compliant mechanisms. Although these were originally created for our own use, we found that others were also interested in their use. Thus began a several-year journey of learning to share information that helped others fabricate FlexLinks themselves. That experience led us to be more intentional in sharing maker information in future projects, and we learned ways to make that process more efficient, impactful, and sustainable. We have found that deciding what to share, where to share it (ranging from email to public repositories), how to communicate where the content is available (ranging from word of mouth to engaging with science influencers), and choosing a sustainable level of engagement are important steps and vary at each level.

### Research

The first level of engagement described is sharing information with research peers, which can be done through email, shared folders, lab websites, or supplemental materials for publications. These are readily available processes but still require researchers to be intentional about sharing.

We first demonstrated FlexLinks in conference presentations and personal meetings, and research peers recognized their value and requested copies for their labs. It was unsustainable to supply hardware, so we began sharing open-source files for peers to construct or modify their own components. As we became more intentional about sharing in later projects, we found that posting the files on a widely available repository made them available without responding to each request, helping to make the effort sustainable.

Public repositories are efficient, accessible, and sustainable choices for sharing maker information. Design-sharing repositories provide 3D printable models and instructions for maker projects or technical models for engineers, designers, and animators. Users often share open-source designs under a Creative Commons license which grants copyright permissions for creative and academic work, and hardware creators may consider an open hardware license^10^. Repositories are usually available at no cost, allow metadata to help with searching, and comply with regulatory requirements (accessibility, privacy, copyright, etc.). This has similarities to the FAIR^11^ initiative and principles which support findability, accessibility, interoperability, and reusability of data. Our lab has found Thingiverse.com and Printables.com to be helpful for 3D printed files, and Instructables.com has been a good resource for more diverse fabrication methods, where more detailed instructions are needed.

Part of being intentional about sharing is communicating the existence and location of the information, which with research peers may be as simple as word of mouth or social media posts, but including it in conference presentations or other research communications can also be effective. Jonathan Hopkins at the University of California, Los Angeles (UCLA), uses videos as supplemental material to peer-reviewed publications^12^, and the video description includes links to maker resources in a public repository.

A recent example of our own lab benefiting from research peer shared maker information occurred when we needed a particular type of micropositioner, and commercially available micropositioners did not meet our needs. We found an open-source 3D printable micropositioner design^13^ and modified it to meet our requirements, which was much quicker and more cost efficient than beginning from scratch.

Sharing with researchers can be straightforward for simple cases such as our FlexLinks illustration. Examples of other simple items that can easily be shared are products that require only a single manufacturing process or board design shared between electronics engineering researchers. However, many open-source hardware projects include more complex instrumentation where more than one fabrication type is involved (printed parts plus electronics with software, mechanics, or optics, for example). It is also often the case that the target group of researchers are not technical experts in that area, for example, when an electronics board is not released for other electrical engineers, but to biologists. In these cases, it can be easier to prepare materials for the more general cases (discussed next) than for peer researchers because of the higher requirements on research equipment performance and calibration.

### Applied

The second level of engagement is sharing with people who can use the information in their work, including in education and industry. Sharing beyond research peers usually requires translation to more accessible language and there is an increased expectation for more detailed instructions.

Educators and students increasingly use maker resources in learning activities^14^ including open-source hardware from design-sharing repositories^15^. Maker information shared by scientific researchers can be useful to educators to teach and inspire the next generation of scientists and engineers.

Makerspaces (collaborative workspaces found inside schools, public libraries, or private facilities) provide hands-on learning experiences to students and others through shared access to high-end manufacturing equipment and creative physical or digital prototyping resources. Makerspace activities can complement those of traditional education channels^16^ by using the notion of ‘making’ to facilitate learning. The rapid surge of makerspaces^17^ have been fueled by the availability and affordability of 3D printers, computer-controlled machine tools, laser cutters, and other prototyping tools^18^.

With FlexLinks, educators and people in industry became aware of the designs from seeing them used by researchers and thereby requested access. Having the maker information available in repositories enabled the devices to become teaching tools for courses or modules, such as at Pennsylvania State University, University of Southern Indiana, Xi’an Jiaotong University, and TU Delft, who also extended the concept to an even broader set of components and materials. As we have become more intentional with sharing maker information, we have seen it used by instructors and as part of student projects in primary and secondary education. Jonathan Hopkins at UCLA also uses open-access university-level educational videos (https://www.youtube.com/@TheFACTsofMechanicalDesign) to teach advanced principles using systems that can be demonstrated in 3D-printed materials.

Sharing with industry can provide inspiration that leads to new products, processes, or services that benefit society and provide economic value. The maker environment empowers people to collaborate, create, and turn creative ideas into products, thus accelerating the adoption of new technologies and fabrication practices. For example, sharing FlexLinks helped practitioners in industry to rapidly prototype designs. This led to sharing other resources, and we have found it rewarding to see them inspire new products. Even in cases where a design is protected as intellectual property, maker information can be valuable in helping secure potential licensees or facilitating startups.

Communicating the existence and location of the material is aided by incorporating metadata in repository postings, such as hashtags and keywords. We have found that combining with other news or media about the research is particularly effective in informing people of the resources.

### Society

The third level of engagement is to the broader public. Maker materials and instructions might be useful to citizen scientists, of interest to the general public with scientific curiosity, and even enjoyed by hobbyists. For example, FlexLinks, in addition to the uses noted above, provided a potential item of interest for building-block enthusiasts. Although immediate and explicit benefits to the researchers may not be as obvious when engaging the general public, as with other groups, it can be personally rewarding and contributes to the virtuous cycle.

A sense of ownership and empowerment can arise from those who engage with maker information, their participation can strengthen confidence in science and technology, and these efforts can cultivate a deeper appreciation for the scientific process. By actively involving the public in research and innovation, scientists can foster a more engaged, informed, and supportive society that values the contributions of science.

Although providing maker information may help motivate future engineers and scientists, and aid citizen scientists, expansion to broader society goes beyond a science focus and can transcend silos. For example, in addition to students interested in science and engineering aspects, others may engage in these making activities as a creative outlet or to learn about concepts in a subject-integrated setting.

An especially effective way to share maker information has been in collaboration with science influencers. In one case, the YouTube science channel Veritasium (https://www.youtube.com/watch?v=97t7Xj_iBv0) did a video on our lab’s technology and included a link in the video description, resulting in over a hundred thousand downloads of maker files. More recently, our group has collaborated with engineer and science influencer Mark Rober and we are sharing maker information for items highlighted in the associated video (https://www.youtube.com/watch?v=9c2NqlUWZfo). This also facilitates connections to our lab’s other existing maker resources.

## Other considerations and challenges

In all levels of sharing, it is important to decide what level of interaction is sustainable after sharing. Popular items will likely result in user comments, remixes, and questions, and it is possible that students, educators, professionals, and others will reach out to researchers. Comprehensive and accessible documentation on how to use the materials (parallel to Readme files in software development) may significantly mitigate the need for further or continuous support and there are lessons to be learned from code development resources, such as GitHub, for version control and collaboration.

It is helpful to make prior decisions about how much time to spend, and when, with whom, and how to engage. For example, we have been surprised at the number of requests to help with students’ class and science fair projects, which are too numerous to accommodate. On the other hand, the virtuous cycle is most clearly demonstrated in the contacts received, which include connections to potential research sponsors, intellectual property licensees, and collaborators.

Because maker information is detailed enough for someone else to make the device, sharing this level of information may be considered public disclosure, which could place the information in the public domain and may restrict future patenting rights. In most cases, commercial rights are not of interest, but it is still wise to define a license, such as CC BY, or an open-source hardware license such as CERN OHL 2^19^

Although these are important considerations, the most worrisome challenges are the risks and ethical concerns about providing the public with certain tools and capabilities. Users are likely to have different goals and motivations than the researchers and some may use the information in ways that are not intended or endorsed by the researchers. Sharing information can inadvertently or intentionally lead to misattribution of contributions, especially with successive modifications and postings. While sharing information can have benefits mentioned in this article, it can also enable the distribution of unprofessional, inaccurate, biased, exclusionary, or physically or emotionally dangerous content. While we believe the positive outcomes of sharing maker information outweigh these potential risks, we recommend researchers always consider them and work to mitigate such potential negative effects as possible.

## Conclusion

Sharing maker information with peers accelerates science and builds cooperation, sharing with educators and industry broadens the impact of research by inspiring future scientists and applications, and sharing with general audiences enriches society. While there are some research areas where employing this approach may be obvious, it would be useful to more researchers than have yet considered it. The infrastructure, tools, and culture are in place for researchers to succeed in their efforts to expand the influence of their research.
